# Humanitarian crisis at the Poland–Belarus border: politics is putting migrants at risk

**DOI:** 10.1016/j.lanepe.2021.100285

**Published:** 2021-12-01

## The Lancet Regional Health – Europe

Amidst the political conflict between the European Union (EU) and Belarus, thousands of migrants from the Middle East have been stranded at the Poland–Belarus border in freezing temperatures and dire conditions since November 8, and at least 11 people have died, some due to hypothermia. Polish border guards fired water cannons and tear gas after migrants threw stones and attempted to destroy a fence, and over 100 migrants have been detained after attempting to cross the Belarus border into Poland. The situation is rapidly evolving each day but is expected to last for months. Pushbacks—state measures by which migrants are forced back over a border without consideration of their individual circumstances and without any possibility to apply for asylum—are a violation of human rights under the European Convention on Human Rights. However, the Polish Government recently passed legislation legalising pushbacks, as a measure to protect so-called Polish culture and sovereignty.

This politically motivated humanitarian crisis is widely understood as a retaliation from Belarus President Alexander Lukashenko against a series of sanctions the EU has imposed on his regime since his disputed election victory in 2020. The European Commission and EU leaders have accused Lukashenko of deliberately assisting the trafficking of migrants from the Middle East to the Poland–Belarus border (to enable entry into Europe via Poland), daring the EU to abandon its humanitarian responsibility as the world looks on. Both Lukashenko´s Government and Russia, a major ally of Belarus, have denied their involvement in this crisis.

Although this crisis is politically motivated, it does not relieve EU from its humanitarian responsibilities. While it is not incumbent on Poland and the EU to provide asylum to all the stranded migrants, it is imperative to uphold the safety, dignity, and human rights of migrants. It is important to shift the focus from politics to what should be done now, to move migrants from precarious situations to a safe place where they can be provided with adequate assistance, and where humane solutions can be found according to individuals’ personal situation and needs.

EU leaders are scrambling to strike a balance between protecting external borders and preventing a worsening humanitarian crisis, however, at present it appears that border security is being prioritised over human rights. Measures have been taken to repatriate over 400 migrants to Iraq, restrict (or even ban) citizens of Afghanistan, Iraq, Yemen, Syria to travel from Dubai to Minsk, and stringent airport controls have been implemented across the Middle East. Many migrants have been temporarily moved to a nearby warehouse in Belarusian territory.

The International Organization for Migration, the UN Refugee Agency, and the International Federation of Red Cross and Red Crescent Societies have delivered some emergency aid. However, more monetary contributions are needed to enable the implementation of more wide-reaching measures, such as transporting migrants via airlift to third countries willing to take them until their papers are processed and their status as refugees or asylum seekers is complete.

Although refugees and asylum seekers are protected by the state by international conventions, migrants are not, and therefore it is convenient for countries to shirk the responsibility of dealing with migrants. An urgent call for action is needed to have an international convention for the protection of migrants and this should include all categories of migrants including environmental migrants—ie, people who are forced to leave their home region due to sudden or long-term changes to their local environments. Protection is needed for any individual who is in a vulnerable situation, where life is at risk, regardless of status. The Geneva convention, which established international legal standards for humanitarian treatment in war, is more than 70 years old, and should be revised to include the most vulnerable groups today. At the same time, the lack of real solidarity in hosting refugees, asylum seekers, and migrants across Europe must be addressed as a priority.

Migration issues are always dealt with using legislations, laws, and politics, and the humane and health perspective is overlooked. Additionally, the Migrant Acceptance Index has declined globally and anti-migrant sentiments are growing. It is important to understand that majority of the migrants do not leave their home country and risk their life unless they absolutely must. In particular, women, children, and unaccompanied minors, who are often the last to leave, do so because they have no other options. Since 2014, Missing Migrants Project has recorded 43 344 deaths globally, over 40% of whom died in the Mediterranean Sea or on the European continent. Individuals who survive the journey from their home country, endure extreme temperatures in unhygienic overcrowded camps with food and water scarcity, and lack of access to appropriate health care for a long period of time. At migrant camps, individuals are often exposed to violence by border guards, death, transactional sex, and can be held in custody without access to a lawyer. Such experiences have a lasting impact on the physical and mental health of migrants who manage to survive their journeys.

Both instrumentalising migrants for political purposes and pushbacks are deplorable. A renewed political commitment is needed to address the migrant crisis in Europe and globally.

